# A direct experimental comparison of single-crystal CVD diamond and silicon carbide X-ray beam position monitors

**DOI:** 10.1107/S1600577523005623

**Published:** 2023-07-18

**Authors:** C. Houghton, C. Bloomer, L. Bobb

**Affiliations:** a Diamond Light Source Ltd, Diamond House Harwell Science and Innovation Campus, Oxfordshire OX11 0DE, United Kingdom; Paul Scherrer Institut, Switzerland

**Keywords:** XBPM, diagnostics, diamond, silicon carbide

## Abstract

Single-crystal chemical vapour deposition (CVD) diamond detectors are an established transmissive synchrotron beamline diagnostic instrument used for beam position and beam intensity monitoring. A recently commercialized alternative is silicon carbide (4H-SiC) devices. These have the potential to provide the same diagnostic information as commercially available single-crystal CVD diamond X-ray beam position monitors, but with a much larger transmissive aperture. At Diamond Light Source an experimental comparison of the performance of single-crystal CVD diamond and 4H-SiC X-ray beam position monitors has been carried out. A quantitative comparison of their performance is presented in this paper. The single-crystal diamond and 4H-SiC beam position monitors were installed in-line along the synchrotron X-ray beam path enabling synchronous measurements at kilohertz rates of the beam motion from both devices. The results of several tests of the two position monitors’ performance are presented: comparing signal uniformity across the surface of the detectors, comparing kHz intensity measurements, and comparing kHz beam position measurements from the detectors. Each test is performed with a range of applied external bias voltages. A discussion of the benefits and limitations of 4H-SiC and single-crystal CVD diamond detectors is included.

## Abstract 2

An experimental comparison between 4H-SiC and single-crystal diamond X-ray beam position monitors as synchrotron light sources is given.

## 1. Introduction

Non-intrusive X-ray beam position monitors (XBPMs) have become vital for real-time monitoring of beam position and intensity at synchrotron facilities as beamline users demand smaller and more stable beams. Intrusive X-ray beam position diagnostics such as fluorescent screens cannot be used during experimental data collection due to the low transmission of X-rays through the materials used for these screens. For example, a 20 µm-thick LuAG scintillator has a photon transmission of just 17% at 12.4 keV (Henke *et al.*, 1993 ▸). The demand for higher transmission beam position monitors led to the research and development of single-crystal chemical vapour deposition (CVD) diamond XBPMs due to their excellent transparency to X-ray photons, radiation hardness and high thermal conductivity (Friedl, 1999 ▸). Early experiments with polycrystalline diamond (Shu *et al.*, 1998 ▸; Sakae *et al.*, 1997 ▸; Bergonzo *et al.*, 1999 ▸) paved the way for modern, commercially available single-crystal CVD diamond (sc-diamond) XBPMs with beam position resolutions of nanometres (Griesmayer *et al.*, 2019 ▸). Silicon carbide [4H-SiC (4H-SiC refers to the polytype of silicon carbide)] XBPMs are a recent development (Nida *et al.*, 2019 ▸) with the potential to provide the same benefits as their diamond counterparts with larger usable apertures and, compared with sc-diamond which requires the use of complex and expensive synthesis processes, 4H-SiC can be fabricated at significantly lower costs (Kushoro *et al.*, 2023 ▸).

Current processes for growing sc-diamond are limited to a maximum plate size of around 5 mm × 5 mm (Nad *et al.*, 2015 ▸). However, 4H-SiC does not have the same constraints and can be grown in wafers with diameters up to 150 mm, from which a number of individual 4H-SiC detector plates can be obtained (Nida *et al.*, 2019 ▸). The use of these larger 4H-SiC plates for X-ray diagnostics potentially has a number of advantages for beamlines: firstly, a larger transmissive window would be beneficial when initially commissioning and aligning beamlines, as it becomes easier to transmit the beam to the sample point; secondly, larger X-ray beam sizes could be accommodated without the XBPM becoming a limiting aperture and causing X-ray scatter from the edge of the detector; and, thirdly, an accurate beam position measurement could be obtained for large X-ray beam sizes, where a beam would ordinarily be too large to be fully measured by an sc-diamond detector.

The individual 4H-SiC plates are 362 µm thick. To improve X-ray transmission the plate can have an ultra-thin 2.3 µm-thick ‘window’ etched into the centre of the 4H-SiC substrate. This thin window is 4.0 mm in diameter. It is challenging to uniformly thin the entire 4H-SiC plate to 2.3 µm, but a 2.3 µm window can be reliably etched within a thicker 4H-SiC plate. A 4.0 mm-diameter aperture is sufficient to enable the transmission of the full X-ray beam, which is generally up to a maximum size of ∼1 mm FWHM on most beamlines at Diamond Light Source. The thicker 4H-SiC substrate acts as a ‘frame’ to support this thin membrane. This technique has previously been demonstrated in sc-diamond (Desjardins *et al.*, 2014 ▸); however, so far such thinning of sc-diamond has not been commercialized for synchrotron XBPMs. This 4H-SiC device is capable of passively dissipating the absorbed power from a typical Diamond Light Source monochromated X-ray beam, up to ∼10^12^ photons s^−1^ at 12 keV (Nida *et al.*, 2019 ▸).

**Figure 1:**
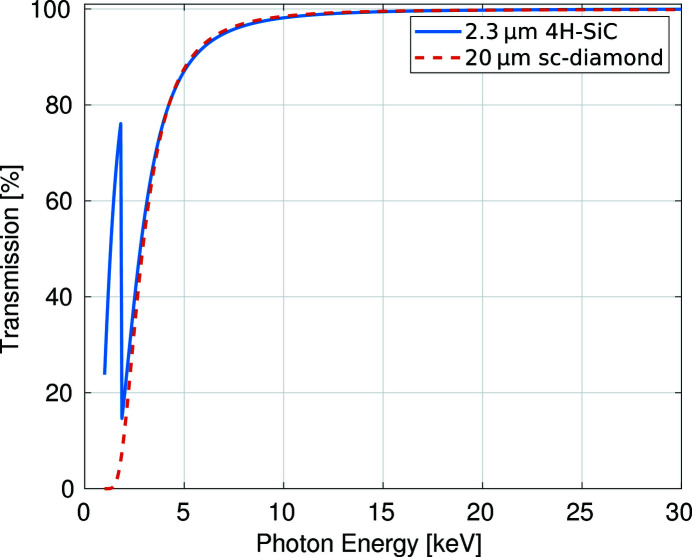
Graph depicting the calculated transmission through both XBPMs for a range of photon energies 1–30 keV based on the cross-section data from Henke *et al.* (1993 ▸).

The two XBPMs used in this experiment were a 362 µm-thick 4H-SiC detector with a 2.3 µm-thick window and a 20 µm-thick sc-diamond detector with uniform thickness across the whole diamond plate. The position-sensitive regions for the devices were 9 mm × 3 mm and 3 mm × 3 mm, respectively. This refers to the region on the device from which it is possible to measure a signal current produced by an X-ray beam. For the 4H-SiC this is both from the thicker 362 µm frame and 2.3 µm-thick central 4.0 mm-diameter window. These two particular detectors and thicknesses are commercially available (the sc-diamond XBPM was purchased from CIVIDEC GmbH, Austria, and the 4H-SiC XBPM was purchased from SenSiC GmbH, Switzerland), and were selected for these tests so that the two detector plates had similar X-ray transmission over the 8–20 keV energy range typically used by beamlines at Diamond Light Source (see Fig. 1 ▸). For example, the following experiments were conducted at 12.4 keV, where transmission is 99.2% and 99.0% for the sc-diamond and 4H-SiC XBPM, respectively (Henke *et al.*, 1993 ▸).

**Figure 2:**
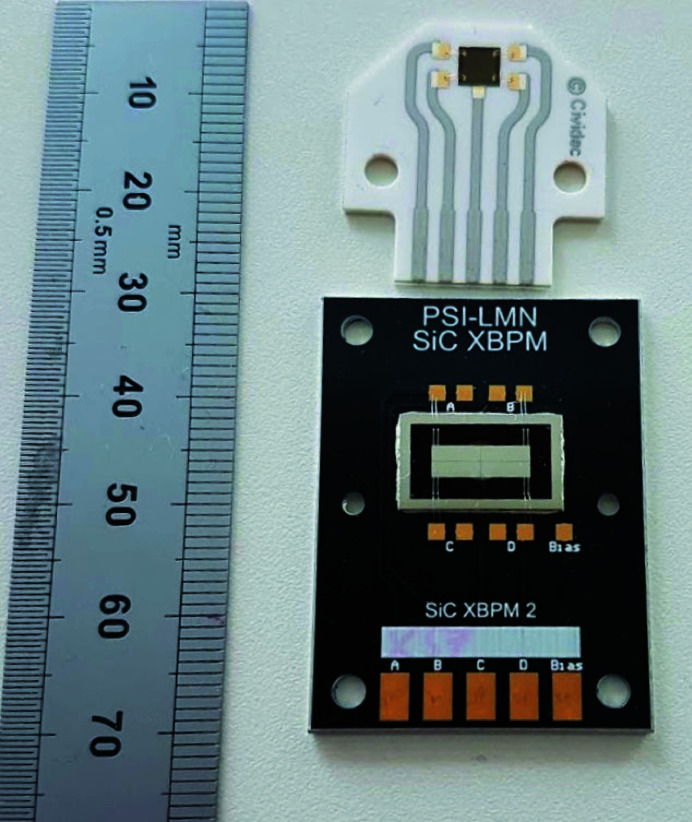
Photograph of the two XBPMs used for this comparison. Top: an XBPM using a 20 µm-thick sc-diamond plate. Bottom: an XBPM with a 362 µm-thick 4H-SiC frame with 2.3 µm-thick window.

An image of the two devices can be seen in Fig. 2 ▸. A previous comparison between two other 4H-SiC and sc-diamond XBPMs was carried out by Houghton *et al.* (2021 ▸) with detector thicknesses of 362 µm and 50 µm, respectively. In that comparison the two thicknesses were chosen again due to their similar X-ray transmission. In that previous 4H-SiC device, the 362 µm-thick region extended across the whole 9 mm × 3 mm position-sensitive area.

Each detector plate is mounted to a printed circuit board (PCB) which features conductive tracks extracting the signal currents, as seen in Fig. 2 ▸. Both PCBs have a hole for the transmission of the photon flux through the device, over which the detector plate is fixed: the sc-diamond XBPM PCB features a 3 mm-diameter hole; the 4H-SiC XBPM PCB features a rounded rectangular 9 mm × 3 mm hole. Ultimately, the transmissive X-ray aperture of the XBPMs is limited to the area of the respective PCB holes.

As is typical for this family of instruments, the detector has four ‘quadrant’ electrodes from which a beam position can be measured (Shu *et al.*, 1998 ▸). The four quadrants are referred to alphabetically such that: *A* = top-left; *B* = top-right; *C* = bottom-right; *D* = bottom-left, when observed from the X-ray source point looking downstream towards the detector.

**Figure 3:**
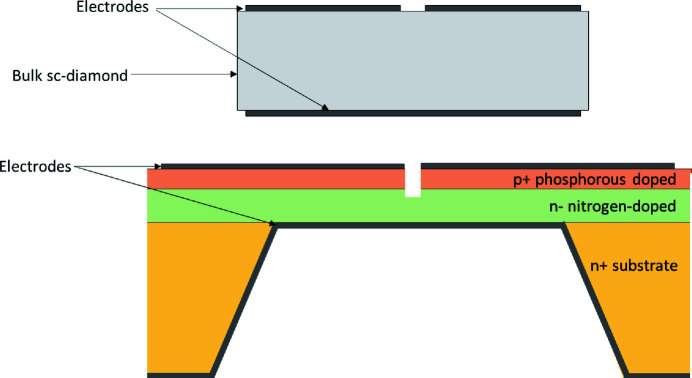
Top: diagram of the cross section of the sc-diamond XBPM. Bottom: diagram of the cross section of the 4H-SiC XBPM, showing the layers of doped 4H-SiC and the thinner central region. (These diagrams are not to scale.)

These ‘quadrant’ electrodes consist of metal contacts applied to the surface of the measurement material, either 100 nm titanium for sc-diamond or 100 nm aluminium 4H-SiC. In both cases, a fifth electrode is applied to the back of the devices to allow a bias voltage to be applied, see Fig. 3 ▸. For the sc-diamond device, the measurement material is solely 20 µm-thick bulk sc-diamond. The 4H-SiC device consists of doped epitaxial layers grown on a 360 µm-thick n-doped substrate. The epitaxial layers consist of a 2 µm-thick n– nitrogen-doped and a 0.3 µm-thick p+ phosphorous-doped 4H-SiC (Medina *et al.*, 2023 ▸; Nida *et al.*, 2019 ▸). The substrate is partially removed creating a thinned-out 2.3 µm-thick central region, leaving the active nitrogen-doped and phosphorous-doped layers. The doped epitaxial layers form a p–n junction.

## 2. Experimental set-up

**Figure 4:**
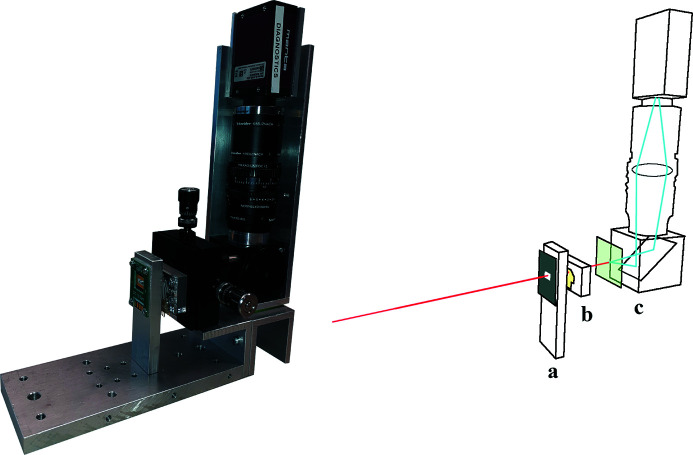
Left: photograph of the assembly used for the direct comparison of the two XBPMs, including the mounting stage. Right: an illustration highlighting the key features of the set-up: **a** – the 4H-SiC XBPM; **b** – sc-diamond XBPM; **c** – fluorescent screen and CMOS camera imaging system.

The experiment was conducted on the I22 beamline at Diamond Light Source (Smith *et al.*, 2021 ▸). The detector mount used to secure the XBPMs during testing is shown in Fig. 4 ▸. The 4H-SiC detector was mounted upstream of the sc-diamond XBPM due to the larger transparent aperture of the 4H-SiC detector. Both devices were each connected to a low-impedance electrometer (TetrAMM; CAENels s.r.l., Italy), capable of recording the current from four channels simultaneously at a rate of 5 kilo-samples per second and outputting a voltage to apply an external bias to the devices, each channel corresponding to one quadrant on the detector’s surface. The intensity of the beam is measured as the summation of the four independently measured channels. Tests were performed in a nitrogen environment and unless specified otherwise an external bias of 5 V was applied to both devices. Downstream of both XBPMs was a fluorescent screen CMOS imaging system, to monitor the transmitted X-ray beam profile for independent verification of beam motion, size and intensity. The three devices were secured to a motorized *X*–*Y* motion stage upstream of the sample point, firstly to enable initial alignment of the detectors to the incident X-ray beam, and secondly to allow the detectors to be raster scanned across the beam.

A focused X-ray beam of horizontal and vertical size σ_*x*_ = 58 µm and σ_*y*_ = 30 µm, respectively, was used to illuminate the detectors. The upstream 4H-SiC XBPM was aligned to the X-ray beam using the assembly’s motorized motion stage so that the signal currents measured were equal on all four quadrants. Then the downstream sc-diamond XBPM was separately aligned to the X-ray beam using a manual *X*–*Y* motion stage (visible in the photograph in Fig. 4 ▸). Following this process, the two devices were aligned to the beam, and the lateral offset between the centres of the two detectors was 8 µm. This was the best achievable alignment that could be obtained using the manual *X*–*Y* stage. Due to the transmission of the 4H-SiC, once aligned the two devices were able to synchronously monitor the same incident beam.

By changing the position of the beam on the detectors using the common motorized *X*–*Y* motion stage and the flux of the beam by use of filters, the response of the devices was quantified. The applied bias voltages could be changed throughout the experiment.

## 3. Results

### 3.1. Spatial uniformity

**Figure 5:**
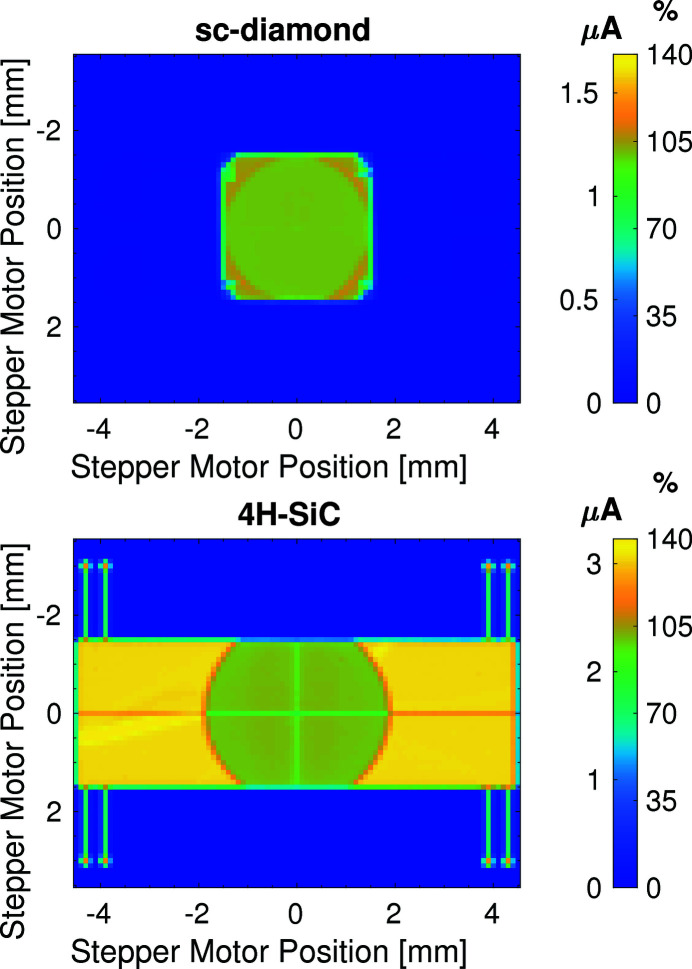
Two-dimensional 9 mm × 7 mm raster scan across the surface of the two XBPMs. Top: 20 µm-thick single-crystal diamond. Bottom: 2.3 µm-thick 4H-SiC within a 362 µm-thick frame.

This comparison aimed to determine whether under the same conditions the two XBPM devices produced similar results, whether this is for spatial uniformity at the centre of the devices, the flux linearity, the temporal response or position measurements. The uniformity of the detector’s response to illumination with X-rays is presented in Fig. 5 ▸. The total signal current measured from each device (*i.e.* the sum of the signal currents from the four quadrants) is presented for each point in the 9 mm × 7 mm two-dimensional raster-scan. The colour bars in Fig. 5 ▸ have two scales: one is the measured signal current in units of µA; the second colour bar scale shows the signal current as a percentage of the average current measured when the X-ray beam is illuminating one of the quadrants (using only the thinner region on the 4H-SiC XBPM for comparable results). The measured current is normalized to remove the effects of storage ring current decay and top-up during the raster scans.

Figure 5 ▸ shows that the ultra-thin region in the centre of the 4H-SiC is visible in the results as a region of weaker signal current. This arises as the ultra-thin (2.3 µm) detector region absorbs fewer photons than the thicker (362 µm) surrounding region, and so less signal current is generated. The larger horizontal size of the 4H-SiC device offers a clear advantage over the sc-diamond XBPM. The vertical size of the active region is the same for both devices.

Also visible is a strip of surface metallization extending from each quadrant of the 4H-SiC device. This strip is used to carry the signal currents closer to the edge of the 4H-SiC plate to reduce the need for long fragile wire bonds. The central transmissive region of the sc-diamond XBPM has high spatial uniformity.

**Figure 6:**
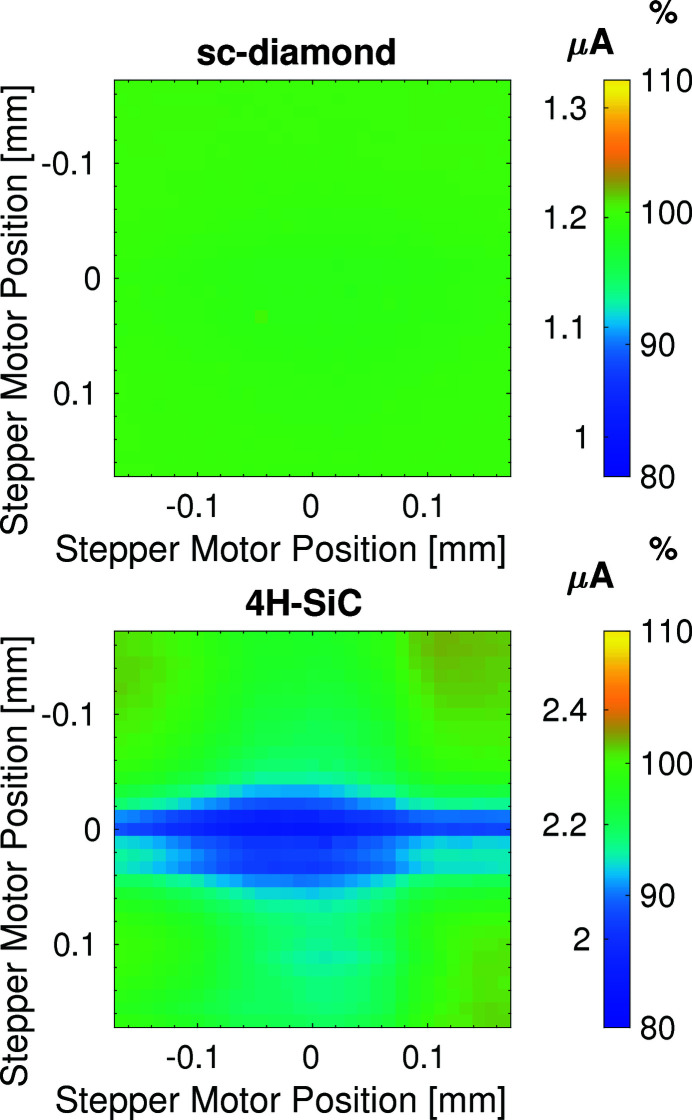
Two-dimensional 0.33 mm × 0.33 mm raster scan across the surface of the two XBPMs. Top: 20 µm-thick single-crystal diamond. Bottom: 2.3 µm-thick 4H-SiC within a 362 µm-thick frame.

The central region of the 4H-SiC XBPM has a gap of 6 µm between the quadrants where a measurable reduction in obtained signal current is observed. The 5 µm gap between quadrants on the sc-diamond device does not result in the same reduction in signal current. The uniformity of the signal across both devices is demonstrated in Fig. 6 ▸ where a finer detail 0.33 mm × 0.33 mm raster scan was completed. At each step in the scan, the signal currents were acquired with an integration period of 0.1 s. The spatial uniformity of the sc-diamond device is excellent with a root-mean-square variation of the signal current of 0.47% across the face of the position-sensitive region of the detector. For the 4H-SiC device the surface current drops to 83.6% of the nominal signal current as the beam passes over the gap between the quadrants, resulting in a root-mean-square variation of the surface current of 4.12%. This reduction in signal could be significant for smaller X-ray beams where a larger percentage of the beam would occupy the gap between quadrants, reducing the signal current, and limiting the device’s use as an intensity monitor. However, as one of the main use cases for the 4H-SiC devices is for relatively large X-ray beams such as the one used in this paper, the impact is minimal as it does not impede the operation for beam position measurements. In addition, once the XBPM is aligned to the beam, any reduction in intensity will be systematic. Information subsequently obtained from the supplier of the 4H-SiC XBPM suggests that this is a consequence of the fabrication techniques used to attach the electrode pads to the surface: the thickness of the detecting region of the 4H-SiC is 25% thinner in the gaps between the quadrants, resulting in a drop in signal current. If the gaps between the quadrants are ignored, the root-mean-square variation was approximately 1.5% on the 4H-SiC, which is about three times greater than the sc-diamond. In more recent developments of the 4H-SiC this reduction in the thickness of the detecting region has been rectified as the p-doped layer is embedded in the n-doped region between quadrants so the thickness is constant across the gap (Medina *et al.*, 2023 ▸).

### 3.2. Bias requirements

External bias voltages are commonly used for solid-state X-ray detectors to increase their sensitivity and accuracy. The bias is used to improve the charge collection efficiency, enabling more charge carriers to be collected at the measurement electrodes. It also increases the net drift velocity of the carriers and thus decreases the magnitude of the lateral diffusion of charge carriers within the bulk material. The requirement for and positive impact of an external bias voltage is well understood for single-crystal diamond detectors (Bohon *et al.*, 2010 ▸; Bloomer & Rehm, 2016 ▸; Di Fraia *et al.*, 2013 ▸; Morse *et al.*, 2007 ▸; Muller *et al.*, 2012 ▸; Bergonzo *et al.*, 1999 ▸).

**Figure 7:**
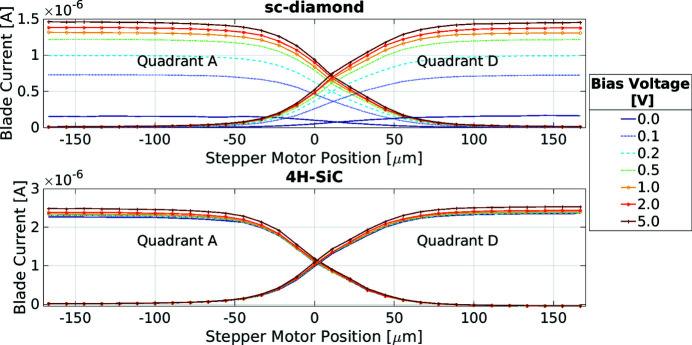
Results of 1D scans of the X-ray beam moving across the face of the detectors, with varying bias voltages applied. Top: 20 µm-thick single-crystal diamond. Bottom: 2.3 µm-thick SiC within a 362 µm-thick frame.

Figure 7 ▸ shows the measured current on two of the quadrants as a one-dimensional stepper motor scan was carried out. This stepper motor scan was repeated with different bias voltages applied to the two XBPMs. The beam moves from quadrant A into quadrant D during the scan. This shows the cross-over between the two quadrants which in both XBPMs is visually symmetrical. The cross-over point differs between the sc-diamond and 4H-SiC devices by ∼8 µm, as the two devices had a small lateral offset and were not perfectly aligned to each other. The step size of the stepper motor scans was 11 µm.

As has been reported elsewhere (Griesmayer *et al.*, 2016 ▸), the sc-diamond XBPM requires a minimum of ∼1 V external bias to achieve a charge collection efficiency above 90% (approximately 50 mV of applied bias per micrometre thickness of diamond). However, the 4H-SiC XBPM needs no applied bias to achieve the same charge collection efficiency. There is little difference between the currents seen with 0 V and 5 V bias voltages applied to the 4H-SiC XBPM, demonstrating that the detector could be run without the need for a bias voltage supply. Unlike the sc-diamond, the 4H-SiC detectors are doped to operate as a p–n junction diode (Nida *et al.*, 2019 ▸). The result is an effective ‘built-in’ electric field that enables the operation of the 4H-SiC device with no externally applied bias voltage. In contrast, due to the difficulty in doping sc-diamond (Koizumi *et al.*, 1994 ▸; Achard *et al.*, 2020 ▸), virtually all commercial sc-diamond detectors require an external bias voltage to be supplied.

### 3.3. Flux linearity

Both sc-diamond and 4H-SiC detector materials use the same fundamental detection mechanism: absorbed ionizing radiation excites electrons from the atomic valance band into the conduction band. The resulting signal current is proportional to the number of charge carriers that are excited, thus proportional to the power absorbed within the detector material. For diamond detectors it has been verified that for a given photon energy the signal current is linear with respect to incident flux over many orders of magnitude (Bohon *et al.*, 2010 ▸).

**Figure 8:**
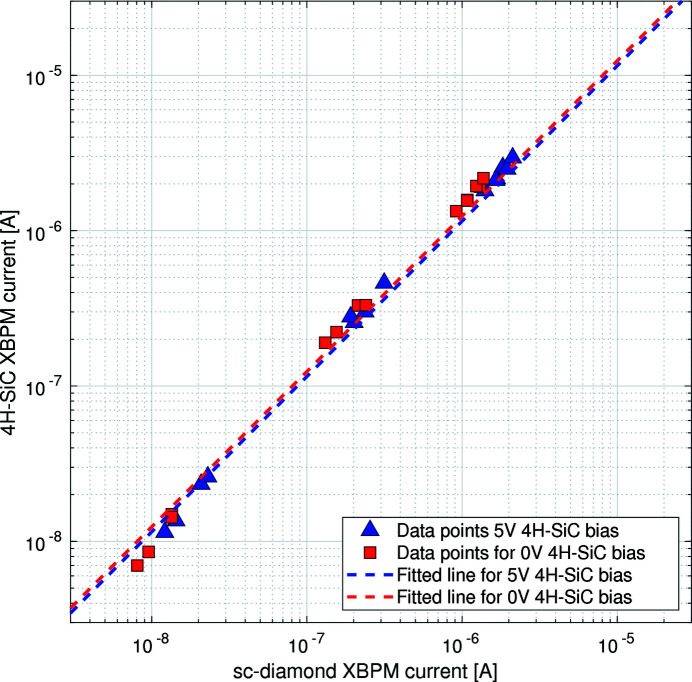
Graph showing the total current measured on the 4H-SiC XBPM with respect to the total current measured on the sc-diamond XBPM as the flux of the incident X-ray beam was changed, with and without a 5 V external bias voltage applied to the 4H-SiC XBPM.

A measurement of the flux linearity of the 4H-SiC detector with respect to the sc-diamond XBPM was carried out (see Fig. 8 ▸). The X-ray beam was attenuated by using various thicknesses of filter material. The blue triangle data-points show the signal current from the 4H-SiC device with a 5 V external bias applied; the red square data-points show the signal current with no external bias applied. The dashed lines represent the linear best fit through each dataset, respectively. The 4H-SiC device shows good linearity with respect to the sc-diamond device. As it is already well established that sc-diamond XBPMs are linear with incident flux, these results demonstrate that this 4H-SiC XBPM is also linear with flux over the evaluated intensity range.

**Figure 9:**
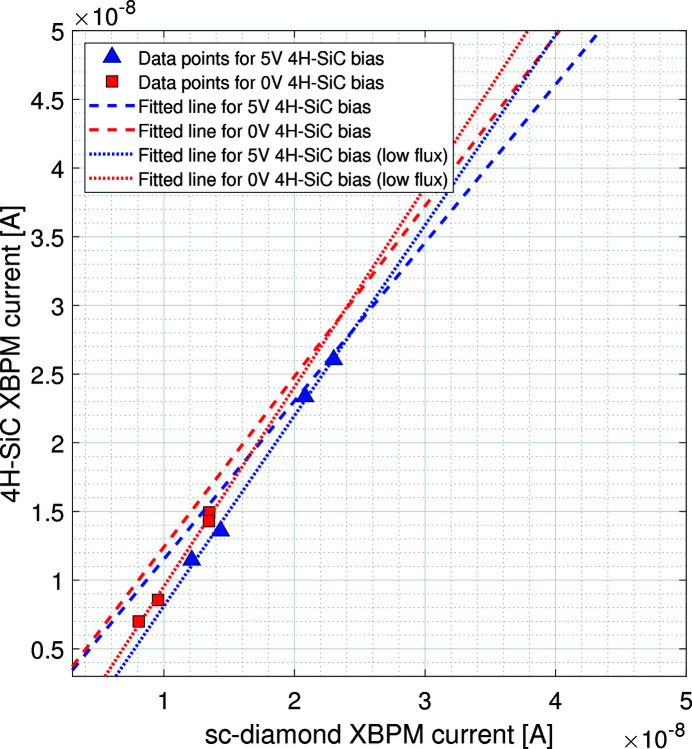
Data from Fig. 8 ▸ focused on the low-flux data showing the total current measured on the 4H-SiC XBPM with respect to the total current measured on the sc-diamond XBPM as the flux of the incident X-ray beam was changed, with and without a 5 V external bias voltage applied to the 4H-SiC XBPM.

There is a deviation from linearity for the 4H-SiC data taken at low flux, see Fig. 9 ▸. The dotted lines show a linear best fit through the low flux data for each dataset. The percentage differences between the gradient of the fit over the full current range and over the low flux range, for the unbiased and biased 4H-SiC datasets, are 17% and 15%, respectively.

### 3.4. Temporal response

**Figure 10:**
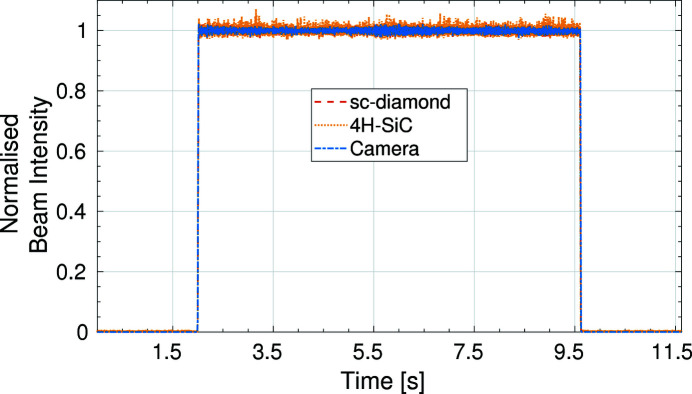
The normalized beam intensity as the experimental hutch shutter is opened and closed for the 4H-SiC and sc-diamond XBPMs and the fluorescent camera imaging system.

An experiment to determine the temporal resolution limitations and the temporal response of the detectors was carried out. The devices were connected to two low-impedance picoammeters capable of collecting data at a rate of 20 kHz for the four signal channels of each XBPM. In addition, beam images were taken using the fluorescent screen imaging system at a rate of 500 Hz. The experimental hutch shutter was opened and closed during a synchronized data acquisition for all three devices. The normalized beam intensity can be seen in Fig. 10 ▸.

**Figure 11:**
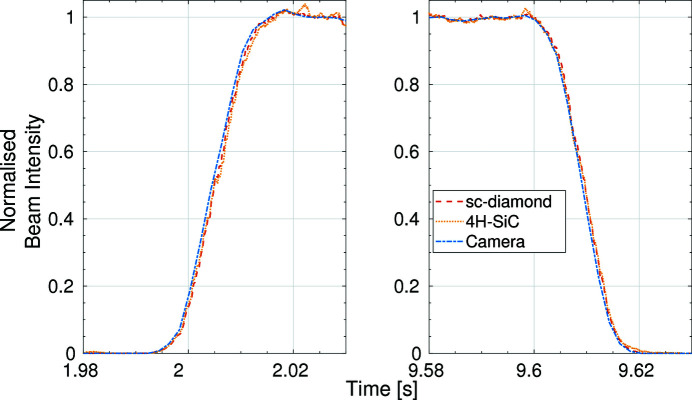
Data from Fig. 10 ▸ focused on the rise times when the shutter was opening (left) and the fall times when the shutter was closing (right).

A more detailed look at the change in intensity as the shutter is opened and closed can be seen in Fig. 11 ▸. It is possible to see small high-frequency variations in the data collected from the XBPMs which could not be resolved on the camera due to the slower acquisition rate. The rise time is the same across all three devices with the normalized beam intensity reaching its maximum within 20 ms, corresponding to the movement time of the shutter.

**Figure 12:**
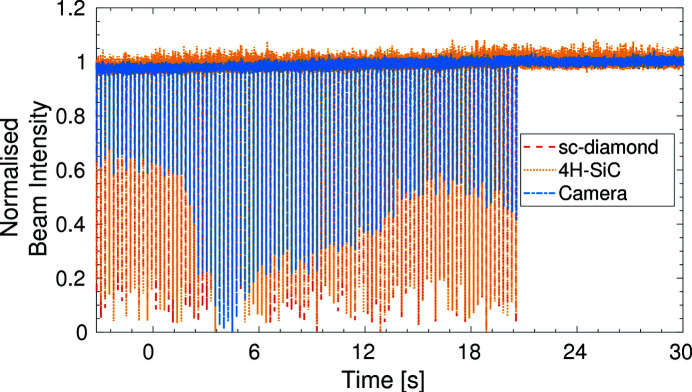
Graph showing the response times for the three devices during the top-up sequence of the synchrotron current.

**Figure 13:**
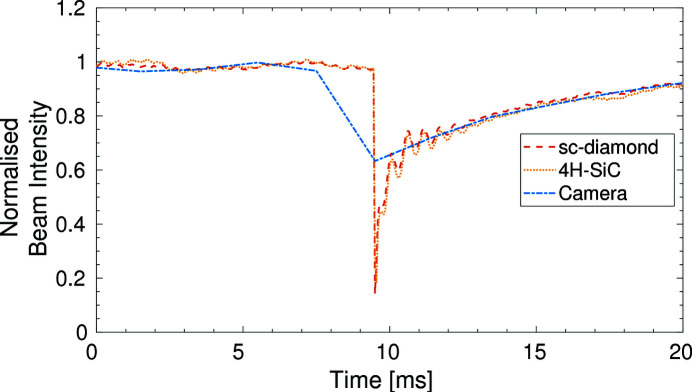
Graph showing the response times for the three devices during the top-up sequence of the synchrotron current, focusing on a 20 ms time period centred on one top-up pulse.

The temporal response of the XBPMs can be further assessed using the synchrotron top-up. Due to electron beam losses, to maintain 300 mA beam current in the storage ring, every ten minutes more electrons are injected into the storage ring at a pulse rate of 5 Hz (Christou *et al.*, 2010 ▸). During this time the X-ray beam size and intensity fluctuate as the stored electron beam is perturbed from the nominal closed-orbit trajectory by an imperfect kicker bump. This motion leads to an increase in emittance due to decoherence which in turn is seen as a measured increase in beam size and reduction in synchrotron radiation intensity on a beamline (Emery & Borland, 1999 ▸). A data collection was set up to purposefully measure the change in intensity of the X-ray beam at the sample point during the synchrotron top-up process. The results of this data collection can be seen in Fig. 12 ▸. Focusing on one of the individual top-up pulses, see Fig. 13 ▸, one can see that the intensity reduction due to the top-up pulse lasts ∼10 ms before returning to the nominal level. It is clear that the limit of the temporal resolution of the camera has been reached and it cannot accurately resolve individual top-up injections. However, both the sc-diamond and 4H-SiC XBPMs see the intensity changes simultaneously during the top-up pulse, and track the same variations in X-ray beam intensity. There is extremely good correlation between the two XBPM intensity measurements, indicating that they both have excellent temporal intensity measurement capabilities at sampling rates of 20 kHz.

### 3.5. Position measurements

**Figure 14:**
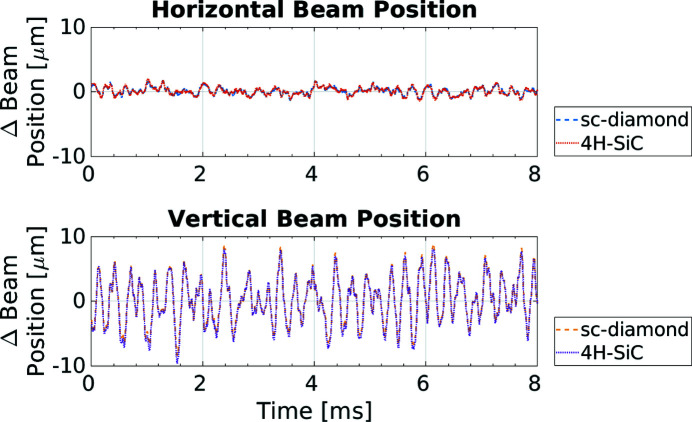
The change in the horizontal (top) and vertical (bottom) beam position from the mean for the 4H-SiC and the sc-diamond XBPMs. An external bias voltage of 5 V was applied to both devices.

The above measurements of the temporal response used data taken when beam variations had been induced by external mechanisms: a shutter and top-up. The following data were taken to replicate user operation to observe the typical beam variations expected during experimental data collection. The position measurements for both XBPM devices are plotted in Fig. 14 ▸. This is the main purpose of these devices. The horizontal and vertical beam positions, *x* and *y*, respectively, can be determined from the XBPM quadrant signals using the difference over sum method, 

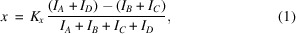




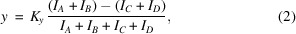

where *I*_(*A*,*B*,*C*,*D*)_ are the currents through the four XBPM quadrants (*A* = top-left; *B* = top-right; *C* = bottom-right; *D* = bottom-left), and *K*_*x*_ and *K*_*y*_ are the scale factors, respectively.

The beam position measurements are almost identical, with any small differences in the measured magnitude of the beam motion likely due to the inaccuracy in the alignment of the two XBPMs with the beam. Therefore the 4H-SiC XBPM can work as a good non-intrusive intensity monitor on beamlines, equally as responsive on ∼1 ms timescales as a traditional sc-diamond XBPM. The root-mean-square variation of beam position as a percentage of beam size was 2.2% and 1.2% horizontally for the sc-diamond and 4H-SiC XBPMs, respectively. Vertically, the variation was larger at 14.86% and 13.84% for the sc-diamond and 4H-SiC XBPMs, respectively.

**Figure 15:**
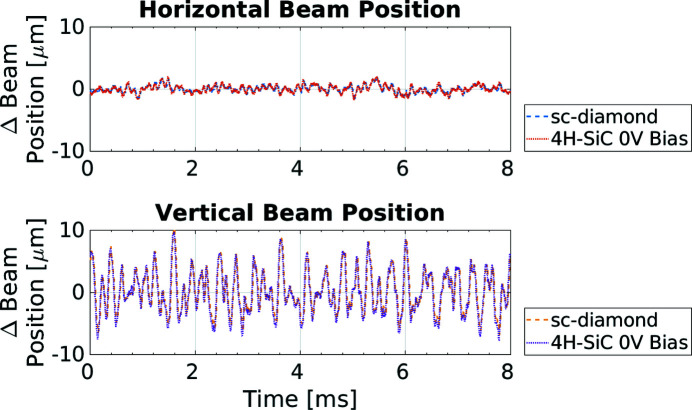
The change in the horizontal (top) and vertical (bottom) beam position from the mean for the 4H-SiC and the sc-diamond XBPMs when there is zero bias applied to the 4H-SiC XBPM.

The synchronous position measurement was repeated, this time with no bias voltage applied to the 4H-SiC XBPM. Again, the position measurement results between the two XBPMs show extremely good correlation. This is shown in Fig. 15 ▸, further corroborating the evidence for the operation of the 4H-SiC XBPM without an external bias voltage.

The position resolution of the detectors is limited by the noise of the detectors and acquisition hardware. The variation in the position measurement is a combination of two components: firstly, the thermal, electrical and quantum noise of the detectors and their cabling; secondly, real beam motion. By only illuminating one quadrant the detectors are not sensitive to real beam position and a measurement of the other noise of the detectors can be obtained. The standard deviation of this position measurement, with the beam fully within one quadrant, can be interpreted as the upper limit of the position resolution. The standard deviation of the measured beam position obtained from the 20 kHz sampling rate on the sc-diamond XBPM was 29 nm and 14 nm for the horizontal and vertical beam positions, respectively. The measured standard deviation on the 4H-SiC XBPM was 187 nm and 76 nm for the horizontal and vertical beam positions, respectively. The standard deviation on beam position is six times larger for the 4H-SiC than the sc-diamond XBPM. Completing the same measurement, illuminating only one quadrant with beam, with no external bias voltage applied to the 4H-SiC XBPM, results in an increase in the measured noise to 243 nm horizontally and 87 nm vertically. It is worth emphasizing that this beam position measurement noise at 20 kHz is still extremely good, and the measured standard deviation of the beam position is still below 0.5% of the beam size (σ_*x*_ = 58 µm and σ_*y*_ = 30 µm).

### 3.6. Transmission

**Figure 16:**
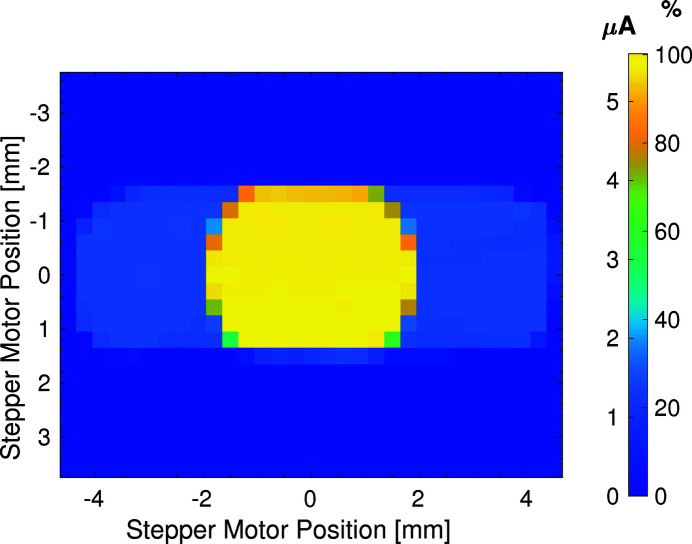
Two-dimensional 4.5 mm raster scan across the surface of a 2.3 µm-thick SiC within a 362 µm-thick frame XBPM. The signal current obtained from an intensity monitoring diode placed downstream of the XBPM assembly is plotted, and the intensity scale shows the diode’s signal current.

The impact of a thin 4H-SiC window within a thicker frame is illustrated in Fig. 16 ▸. This figure presents a measurement of the transmission of 12.4 keV X-ray photons through the device. The sc-diamond device and camera imaging system shown in Fig. 4 ▸ were removed and a fixed diode was placed downstream of the 4H-SiC XBPM. A two-dimensional raster scan of the XBPM was conducted. The current measured on the diode and the current as a percentage of the average current seen on the diode with no upstream devices is shown in Fig. 16 ▸. The thicker region of the 4H-SiC plate still allows 20% of the X-ray photons to be transmitted further down the beamline to the diode. The large size of this semi-transmissive region is beneficial for the beamline and aids in the initial coarse alignment of the X-ray beam. The requirements for accurate initial alignment of the XBPM are relaxed, as useful beam position information can be obtained even for relatively large misalignments, and some beam can reach other downstream diagnostics which is helpful for commissioning purposes. A horizontal misalignment larger than 1.5 mm would result in the X-ray beam completely missing the position-sensitive region of the smaller sc-diamond XBPMs. The larger position-sensitive region of the 4H-SiC XBPM over the sc-diamond XBPM makes initial alignment and commissioning easier for beamline staff, improving the speed at which the beamline can be set up for users and helping to reduce beamline down-time.

## 4. Conclusion

Through comparative simultaneous testing of commercially available sc-diamond and new 4H-SiC XBPMs, it was found the 4H-SiC XBPM has the equivalent operational performance to the sc-diamond XBPM in beam position measurement and flux linearity with high flux. The very thin 4H-SiC XBPMs are shown to have slightly worse spatial uniformity compared with the 20 µm-thick sc-diamond XBPM, most likely due to inhomogeneities in the detector plate thickness. Small variations in the surface roughness are more noticeable when the detector plate is only 2.3 µm thick. A signal drop of 15% in the 6 µm gap between quadrant electrodes with a beam size of σ_*x*_ = 58 µm, σ_*y*_ = 30 µm could reduce the effectiveness of the 4H-SiC device as an intensity monitor especially for smaller X-ray beam sizes as a larger proportion of X-rays will fall in the gaps. However, due to the ability of 4H-SiC to be produced with larger apertures, the applications would involve larger beams where the gap would have less of an impact. For this application, this reduction in signal current does not negatively impact the device’s use as a beam position monitor. The 4H-SiC XBPMs can be operated without an external bias voltage over the photon flux range examined in these tests, simplifying the installation on beamlines. Operating without an applied bias voltage has negligible impact on the beam position measurement.

In addition, the 4H-SiC can be produced with a larger transmissive aperture with a thinner central region allowing for 99% transmission and a thick 362 µm-thick region providing 20% transmission. This is beneficial for beamlines with larger beam sizes and in the alignment of the X-ray beam where the use of the smaller 3 mm transmissive aperture provided by the sc-diamond XBPM would limit performance.

Through appropriate choice of 4H-SiC thickness, 4H-SiC detectors can offer comparable transmission through the central region to sc-diamond XBPMs for *in situ* beam position measurements.
